# Odontogenic Maxillary Sinusitis With Sequestrum-Like Bone Formation Mimicking Fungal Sinusitis: A Case Report

**DOI:** 10.7759/cureus.112498

**Published:** 2026-07-11

**Authors:** Yoshiki Sato, Shintaro Yodogawa, Masaki Tamura, Hiroki Nagayasu, Tsuyoshi Shimo

**Affiliations:** 1 Department of Human Biology and Pathophysiology, Division of Reconstructive Surgery for Oral and Maxillofacial Region, School of Dentistry, Health Sciences University of Hokkaido, Hokkaido, JPN

**Keywords:** aspergillosis, bone formation, calcification, fungal sinusitis, maxillary sinus, odontogenic maxillary sinusitis, sequestrum

## Abstract

Calcified materials in an opacified maxillary sinus often suggest fungal sinusitis, particularly aspergillosis. However, odontogenic maxillary sinusitis may rarely be associated with a bone-like calcified mass, creating diagnostic difficulty. We report a case of chronic odontogenic maxillary sinusitis with sequestrum-like bone formation that mimicked fungal sinusitis. An 88-year-old man was referred for mobility and occlusal pain of the maxillary left first molar. He had no nasal obstruction, rhinorrhea, headache, facial pain, or history of maxillary sinus surgery. Intraoral examination showed marked periodontal destruction, root exposure, tooth mobility, and percussion and occlusal pain. A hard structure was palpable through the periodontal pocket. Panoramic radiography showed increased opacity of the left maxillary sinus and a well-defined, horseshoe-shaped, bone-like radiopaque mass near the root apex. Computed tomography revealed a capsular calcified structure adjacent to the root apex within soft tissue occupying the left maxillary sinus, with extension toward the ethmoid sinus and thickening of the sinus wall. Fungal maxillary sinusitis was considered in the radiographic differential diagnosis.

Under local anesthesia, extraction of the affected tooth, removal of the calcified mass, and maxillary sinus fenestration were performed. The mass, identified through the extraction socket, was not adherent to the tooth and was easily separated from the surrounding tissue. No fungal organisms, including Aspergillus species, were identified on histopathological examination. The excised specimen was a hard bone-like mass measuring approximately 20 × 15 mm. Histopathological examination revealed a sequestrum with areas suggestive of pre-existing lamellar bone and newly formed, incompletely mineralized bone. At one-year follow-up, maxillary sinus mucosal thickening had improved. This case illustrates that odontogenic maxillary sinusitis may be associated with a bone-like calcified mass and may radiographically mimic fungal sinusitis. When surgical treatment is clinically indicated, histopathological examination of the removed lesion can help establish a definitive diagnosis.

## Introduction

Odontogenic sinusitis is a distinct form of sinonasal disease caused by dental infection, periodontal disease, oroantral communication, or dental treatment-related factors. Its etiology, microbiology, diagnostic approach, and treatment strategy differ from those of non-odontogenic rhinosinusitis. Therefore, recognition of the dental source is essential for appropriate management [1,2]. In particular, unilateral maxillary sinus opacification should prompt careful evaluation of adjacent maxillary teeth and periapical or periodontal lesions, because odontogenic infection is a major cause of unilateral maxillary sinusitis [3,4]. From a multidisciplinary diagnostic perspective, integration of sinonasal evaluation by otolaryngologists and dental assessment of odontogenic pathology is important for accurate diagnosis and management [5].

Calcified or hyperdense materials within an opacified maxillary sinus often raise suspicion for fungal sinusitis, especially a maxillary sinus fungus ball. Intralesional hyperdensity or calcification on computed tomography is widely regarded as an important radiographic clue for fungal sinusitis [6]. However, calcified or bone-like materials in the maxillary sinus are not specific to fungal disease and may also be associated with odontogenic inflammation, antrolith formation, isolated bone fragments, or ectopic bone formation.

Here, we report a case of chronic odontogenic maxillary sinusitis associated with the maxillary left first molar, presenting with a bone-like calcified mass in the left maxillary sinus. The lesion clinically and radiographically mimicked fungal sinusitis, but histopathological examination revealed a sequestrum with areas suggestive of newly formed, incompletely mineralized bone. This case highlights a diagnostic pitfall in the interpretation of calcified maxillary sinus lesions and emphasizes the importance of clinicoradiographic correlation and histopathological confirmation.

## Case presentation

This retrospective case involved an 88-year-old man who was referred to our department by a dental clinic in January 2011 because of mobility and occlusal pain of the maxillary left first molar and panoramic radiographic findings suggestive of left maxillary sinusitis with a bone-like radiopaque mass near the root apex of the affected tooth. His medical history included heart failure, bronchial asthma, and hypertension, and his family history was unremarkable. He had no nasal obstruction, rhinorrhea, headache, facial pain, or history of previous maxillary sinus surgery or intrasinus treatment. Nasal endoscopy was not performed at the initial evaluation, and detailed assessment of the middle meatus or osteomeatal complex was not available in the retrospective records.

Extraoral examination showed a symmetrical facial appearance, with no swelling, tenderness, or sensory disturbance in the left cheek. No marked nasal obstruction or nasal discharge was observed, and blood examination revealed no remarkable abnormalities. Intraoral examination showed marked periodontal destruction around the maxillary left first molar. The tooth showed horizontal and vertical mobility, percussion pain, and occlusal pain. Severe alveolar bone resorption was observed, and the root was exposed almost to the apex (Figures 1A, 1B). When a dental probe was inserted through the periodontal pocket, a hard bone-like structure was palpable. Panoramic radiography showed increased opacity of the left maxillary sinus and a well-defined bone-like radiopaque mass adjacent to the root apex of the maxillary left first molar (Figure 1C). No obvious bone destruction was observed. Computed tomography showed a capsular calcified structure adjacent to the root apex of the maxillary left first molar within soft tissue occupying the left maxillary sinus (Figure 1D). The structure had a computed tomography value of approximately 200 Hounsfield units. The soft tissue extended toward the ethmoid sinus, and thickening of the sinus wall was also observed. Based on these clinical and radiographic findings, the clinical diagnosis was chronic odontogenic maxillary sinusitis arising from chronic suppurative apical periodontitis of the maxillary left first molar, with fungal maxillary sinusitis considered in the radiographic differential diagnosis.

**Figure 1:**
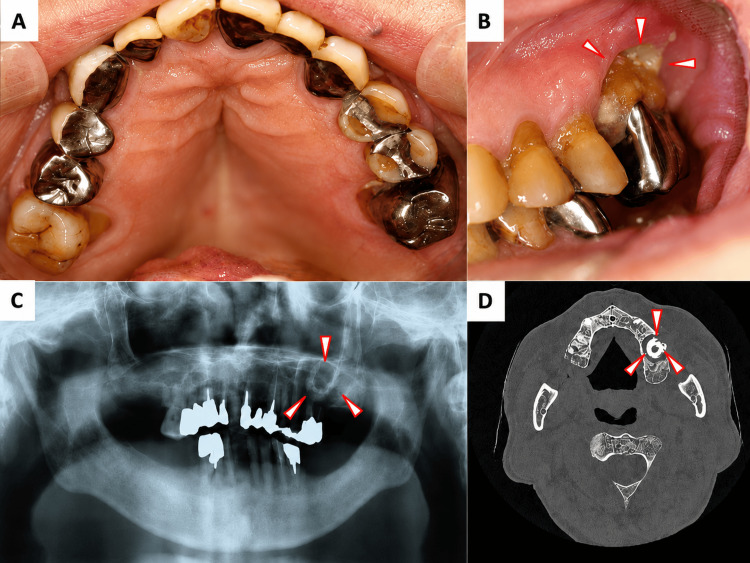
Initial intraoral, panoramic radiographic, and computed tomography findings. (A) Palatal intraoral photograph showing the maxillary oral cavity at the first visit. (B) Lateral intraoral photograph showing severe periodontal destruction and root exposure around the maxillary left first molar. The red arrows indicate mucosal swelling and the affected molar region. (C) Panoramic radiograph showing increased opacity of the left maxillary sinus and a well-defined bone-like radiopaque mass. The red arrowheads indicate the radiopaque lesion adjacent to the root apex of the maxillary left first molar. (D) Axial computed tomography image showing a calcified structure adjacent to the root apex of the maxillary left first molar within the left maxillary sinus. The red arrowheads indicate the calcified lesion.

On February 27, 2011, extraction of the maxillary left first molar, removal of the calcified mass from the left maxillary sinus, and maxillary sinus fenestration were performed under local anesthesia. The ethmoid sinus was not surgically addressed. The soft tissue extension toward the ethmoid sinus on preoperative computed tomography was considered secondary inflammatory mucosal involvement associated with the maxillary sinus lesion. The calcified mass was identified through the extraction socket. No adhesion was observed between the affected tooth and the calcified mass. The mass was easily separated from the surrounding tissue and removed. Inflamed soft tissue within the maxillary sinus was also removed through the extraction socket, and the surgical site was left open for drainage (Figures 2A, 2B). The removed calcified mass and inflamed soft tissue were submitted for histopathological examination.

**Figure 2:**
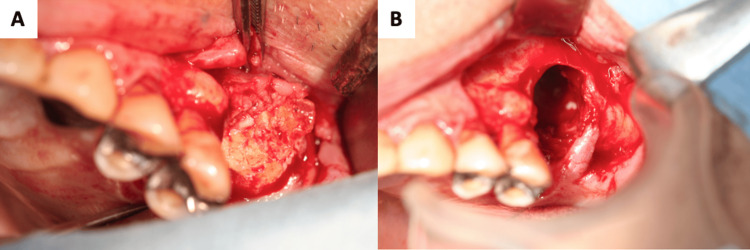
Intraoperative findings during removal of the calcified mass. (A) The calcified mass was exposed through the extraction socket and was easily separated from the surrounding tissue. (B) After removal of the calcified mass from the maxillary sinus through the extraction socket, the surgical site was left open for drainage.

The excised specimen was a hard bone-like mass measuring approximately 20 × 15 mm (Figures 3A, 3B). Histopathological examination showed necrotic bone with inflammatory cell infiltration, consistent with sequestrum. Bacterial culture was not performed, and no specific bacterial species was isolated. Another area showed a transition between pre-existing lamellar bone and newly formed bone-like tissue, with incomplete mineralization toward the newly formed component (Figures 3C, 3D). No fungal organisms, including Aspergillus species, were identified on histopathological examination. KOH preparation and fungal culture were not performed in this retrospective case. The final pathological diagnosis was sequestrum.

**Figure 3:**
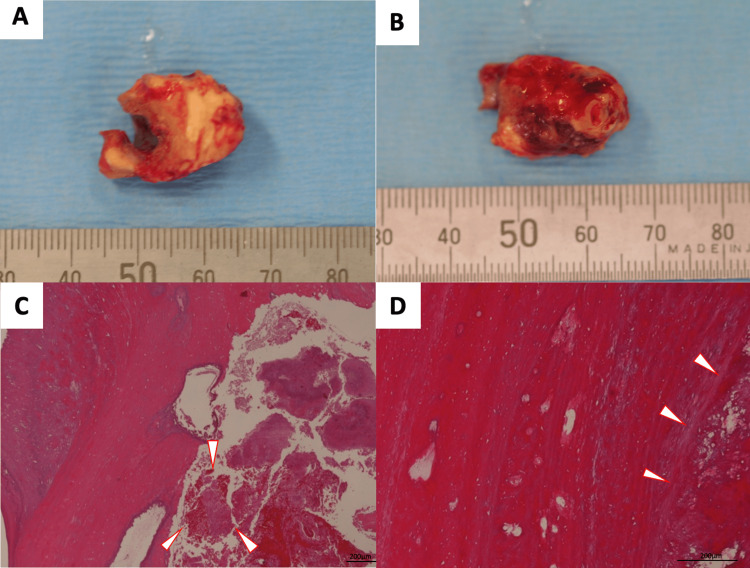
Gross and histopathological findings of the excised calcified mass. (A, B) Gross photographs showing the excised hard bone-like mass, which measured approximately 20 × 15 mm. (C) Low-power H&E-stained photomicrograph showing necrotic bone consistent with sequestrum. The arrows indicate necrotic bone consistent with sequestrum. Scale bar: 200 μm. (D) Higher-power H&E-stained photomicrograph showing areas of incomplete mineralization, supporting the presence of newly formed bone that subsequently became necrotic. The arrows indicate newly formed, incompletely mineralized bone. Scale bar: 200 μm.

The postoperative course was uneventful. Because the surgical site was left open for drainage, postoperative care focused on preventing food impaction and regurgitation into the maxillary sinus. The patient was instructed to avoid forceful rinsing and nose blowing and was followed carefully for signs of food regurgitation, nasal leakage, or sinus infection. No such complications were observed. At one-year follow-up in March 2012, mucosal thickening of the left maxillary sinus had improved. The patient was managed with an obturator prosthesis and had a favorable clinical course without speech disturbance (Figures 4A-4E). No additional long-term follow-up data beyond March 2012 were available, and the patient was not under active follow-up at our department at the time of manuscript preparation.

**Figure 4:**
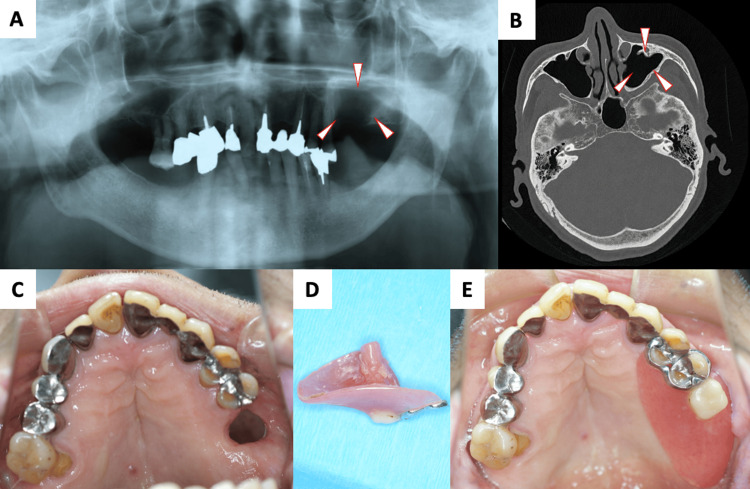
Postoperative findings at one-year follow-up. (A) Panoramic radiograph showing improvement of opacity in the left maxillary sinus after surgery. The red arrowheads indicate the postoperative left maxillary sinus region. (B) Axial computed tomography image showing improvement of mucosal thickening in the left maxillary sinus. The red arrows indicate the improved left maxillary sinus region. (C) Intraoral photograph showing the postoperative condition of the extraction and fenestration site. (D) Photograph of the obturator prosthesis used for postoperative functional rehabilitation. (E) Intraoral photograph showing the obturator prosthesis in place. The patient had a favorable postoperative course without speech disturbance.

## Discussion

The present case was characterized by a bone-like, calcified mass in the left maxillary sinus, associated with chronic odontogenic maxillary sinusitis arising from the left maxillary first molar. Although the overall clinical and radiographic findings favored odontogenic maxillary sinusitis, the calcified structure within the opacified maxillary sinus raised the possibility of fungal maxillary sinusitis in the radiographic differential diagnosis. However, no fungal organisms, including Aspergillus species, were identified on histopathological examination; neither a KOH preparation nor a fungal culture was performed in this retrospective case. Histopathological examination instead revealed sequestrum with areas suggestive of pre-existing lamellar bone and newly formed, incompletely mineralized bone. These findings indicate that the calcified mass was not a fungal-related concretion but a sequestrum-like bone mass that developed in the setting of chronic odontogenic inflammation.

Several clinical and radiographic features supported an odontogenic origin in this case. The maxillary left first molar showed severe periodontal destruction, marked mobility, percussion pain, and occlusal pain. The root was exposed almost to the apex, and a hard structure was palpable through the periodontal pocket. Radiographically, the calcified lesion was located adjacent to the root apex of the affected tooth, and intraoperatively, the lesion was identified and removed through the extraction socket. These findings suggest a close anatomical and pathological relationship between the diseased maxillary molar and the maxillary sinus lesion. Odontogenic sinusitis is often unilateral and may arise from periapical infection, periodontal disease, oroantral communication, or previous dental treatment [1-4]. In the present case, severe periodontal destruction of the maxillary left first molar was considered the most likely source of chronic odontogenic inflammation.

The international multidisciplinary consensus statement on diagnosing odontogenic sinusitis emphasizes that otolaryngologists generally confirm sinusitis based mainly on nasal endoscopic findings, such as middle meatal purulence, edema, or polyps, whereas dental providers confirm maxillary odontogenic pathology through clinical examination and dental imaging [5]. In the present case, the odontogenic source was strongly supported by dental examination, panoramic radiography, computed tomography, intraoperative findings, and histopathological examination. However, nasal endoscopy was not performed, and a detailed assessment of the middle meatus or osteomeatal complex was unavailable, which represents a limitation of this retrospective case.

Although the clinical and radiographic findings strongly suggested odontogenic maxillary sinusitis, the calcified appearance within the opacified maxillary sinus raised the radiographic differential diagnosis of fungal maxillary sinusitis. Calcification or intralesional hyperdensity within an opacified maxillary sinus is a well-known radiographic clue for a maxillary sinus fungus ball [6]. Yamauchi et al. reported that intrasinus calcification was frequently detected in non-invasive chronic fungal maxillary sinusitis [7]. Broglie et al. described sinus opacification, with or without flocculent calcifications, as an important radiological finding of sinus fungus ball and reported that preoperative computed tomography had limited positive predictive value [8]. In addition, Alharbi et al. described computed tomography hyperdensities in more than 60% of paranasal sinus fungus ball cases [9].

Yoon et al. showed that the pattern of intrasinus calcification on computed tomography could be useful for differentiating fungal from non-fungal chronic maxillary sinusitis [10]. Cha et al. further emphasized that intralesional hyperdensity alone is not sufficient for a definitive preoperative diagnosis and should be interpreted together with other clinical and radiographic findings [11]. Thus, fungal maxillary sinusitis was considered because of the calcified or hyperdense appearance within the opacified maxillary sinus; however, the overall clinical and radiographic findings favored an odontogenic origin. Nevertheless, no fungal organisms, including Aspergillus species, were identified on histopathological examination, and the pathological diagnosis of sequestrum indicated that the calcified lesion in this case was not a fungal-related concretion. The intralesional hyperdensity of a fungus ball is generally attributed to the deposition of calcium salts and heavy metals within the fungal mass, and its computed tomography density has been described in both lower-density (organic) and higher-density (inorganic) patterns [9]. In the present case, the lesion showed a relatively low computed tomography value of approximately 200 Hounsfield units and was histopathologically composed of necrotic and newly formed bone rather than mycotic concretions, which further supports that the calcified mass represented sequestrum-like bone formation rather than a fungal-related calcification.

Calcified lesions in the maxillary sinus may arise from several different pathological processes. In addition to fungal sinusitis, the differential diagnosis includes antrolith, isolated bone fragment, ectopic bone formation, osteoma, foreign body-associated calcification, displaced root fragment, and sequestrum. Borrás-Ferreres et al. reported odontogenic maxillary sinusitis and fungus ball development secondary to a dental root retained in the maxillary sinus for more than 25 years [12]. Ogura et al. reported odontogenic maxillary sinusitis with many calculi and suggested that small sequestra in the sinus membrane may have acted as nuclei for stone formation [13]. Antroliths have also been described as rare calcified masses within the maxillary sinus [14,15]. These reports are similar to the present case in that inflammatory maxillary sinus disease was associated with calcified material. However, the previously reported lesions were retained dental root-related fungus balls or antroliths, whereas the present case involved a single hard bone-like mass measuring approximately 20 × 15 mm. The clinical characteristics, lesion size, preoperative diagnosis, treatment, postoperative histopathological diagnosis, fungal detection status, and outcomes of relevant reported cases are summarized in Table 1. Available radiological characteristics, including CT appearance and attenuation values when reported, are also described in the table legend.

**Table 1:** Reported maxillary sinus lesions with calcified or osseous materials relevant to the present case. This table summarizes previously reported maxillary sinus lesions with calcified or osseous materials and compares them with the present case. Available radiological characteristics were reviewed from each report. CT attenuation values were not consistently reported in the previous cases listed in the table. In the present case, the lesion appeared as a capsular, calcified mass adjacent to the root apex of the left maxillary first molar, with an attenuation of approximately 200 Hounsfield units. F: female; M: male; NR: not reported

Studies	Age	Sex	Background	Lesion	Size/number	Preoperative clinical diagnosis	Treatment	Postoperative histopathological diagnosis	Aspergillus/fungal organisms detected	Outcome
Ogura et al. (2003) [13]	37	F	Odontogenic sinusitis	Multiple calculi/antroliths	55 calculi, 1-5 mm	Odontogenic maxillary sinusitis with calcified masses	Extraction of the affected molar and maxillary sinusectomy	Maxillary sinusitis with many calculi; small sequestra in the sinus membrane	NR	NR
Shenoy et al. (2013) [14]	47	F	Recurrent sinusitis with loosening of the left maxillary second molar	Maxillary antrolith	20 × 10 mm	Recurrent maxillary sinusitis with oroantral fistula; suspected osteoma or malignant lesion	Endoscopic sinus surgery, antrostomy, and Caldwell-Luc procedure	Maxillary antrolith	No	Symptoms improved after surgical removal
Tan et al. (2020) [15]	67	F	Incidental radiographic finding	Maxillary antrolith	3.1 × 3.6 mm	Maxillary antrolith	Caldwell-Luc procedure and excisional biopsy	Metaplastic bone and calcification, consistent with maxillary antrolith	NR	No recurrence after removal
Higuchi et al. (2023) [16]	24	M	Suspected displaced root fragment after extraction	Multiple isolated bone fragments	Multiple fragments; individual size NR	Right maxillary sinusitis with suspected foreign bodies or multiple isolated bone fragments	Removal of right maxillary sinus foreign bodies/bone fragments and extraction of the right maxillary first molar	Multiple isolated bone fragments	NR	Pain resolved at one year
Sato et al. (present case)	88	M	Odontogenic sinusitis from the maxillary left first molar	Single bone-like mass	Approximately 20 × 15 mm	Chronic odontogenic maxillary sinusitis from the maxillary left first molar; fungal maxillary sinusitis considered radiographically	Extraction of the maxillary left first molar, removal of the calcified mass, and maxillary sinus fenestration	Sequestrum with areas suggestive of newly formed, incompletely mineralized bone	No	Sinus mucosal thickening improved at one year

This comparison highlights several features that distinguish the present case from previously reported calcified or osseous maxillary sinus lesions. First, the present lesion was a single relatively large bone-like mass, whereas previous reports mainly described multiple small calculi, antroliths, or isolated bone fragments. Second, fungal maxillary sinusitis, including aspergillosis, was considered preoperatively because the lesion showed a calcified or hyperdense bone-like structure within an opacified unilateral maxillary sinus, which is a known radiographic feature of a maxillary sinus fungus ball. However, the odontogenic origin was strongly suggested by the diseased left maxillary first molar and the close anatomical relationship between the calcified mass and the root apex. No fungal organisms, including Aspergillus species, were identified on histopathological examination. Third, the final diagnosis was sequestrum with areas suggestive of newly formed, incompletely mineralized bone, rather than antrolith, fungus ball, or osteoma. Finally, the lesion was removed through the extraction socket together with treatment of the odontogenic source, which allowed both diagnosis and management without a more extensive surgical approach. These differences suggest that calcified lesions in an opacified maxillary sinus may be interpreted in relation to the adjacent teeth, operative findings, fungal detection status, and histopathological features.

The present case also overlaps with previously reported maxillary sinus lesions involving antroliths or isolated bone fragments. Higuchi et al. reported multiple isolated bone fragments in the maxillary sinus that were initially suspected to be displaced root fragments after tooth extraction [16]. Cho et al. evaluated maxillary antroliths detected by cone-beam computed tomography in an adult dental population and showed that these lesions were rare and usually asymptomatic [17]. Manning et al. reported a chronic sinusitis-associated antrolith, and Nair et al. reported an antrolith in the maxillary sinus with a bony nidus after endoscopic sinus surgery [18,19]. These reports support the concept that calcified or bone-like structures can exist within the maxillary sinus and may be difficult to distinguish from displaced roots, foreign bodies, antroliths, fungal-related calcifications, or inflammatory bone-like lesions on imaging alone.

Other osseous lesions may also be considered in the differential diagnosis. Viswanatha described maxillary sinus osteoma as a benign osteogenic lesion that may appear as a bone-like radiopacity within the maxillary sinus [20]. In the present case, the lesion was easily separated from the surrounding soft tissue, was not adherent to the left maxillary first molar, and was histologically diagnosed as a sequestrum rather than an osteoma. These intraoperative and pathological findings were useful for excluding other osseous lesions.

The odontogenic origin of the maxillary sinusitis in this case was strongly supported by the clinical, radiographic, intraoperative, and histopathological findings. However, the precise mechanism by which the sequestrum-like bone mass formed remains speculative, and several mechanisms may be considered. Chronic inflammation from the maxillary left first molar may have extended into the maxillary sinus and induced persistent inflammation of the sinus mucosa and adjacent periosteal tissues. Repeated inflammatory stimulation can promote bone remodeling, local osteogenesis, and secondary necrosis. In the present case, histopathological examination showed necrotic bone consistent with a sequestrum, as well as areas suggestive of pre-existing lamellar bone and newly formed, incompletely mineralized bone. These findings suggest that the lesion may not have been a simple detached bone fragment. Rather, local bone formation may have occurred in association with chronic odontogenic inflammation, followed by necrosis and separation as a sequestrum-like mass.

This interpretation is supported by the relationship between the lesion and the diseased tooth. The calcified mass was adjacent to the root apex of the maxillary left first molar, and a hard structure could be palpated through the periodontal pocket. During surgery, the mass was identified through the extraction socket and was removed together with inflamed sinus tissue. Although the mass was not adherent to the tooth, the spatial relationship between the two supports an odontogenic inflammatory process. Taken together, the association with clear odontogenic inflammation, the presence of both necrotic and newly formed bone, and the radiographic mimicry of fungal sinusitis within an opacified maxillary sinus suggest that similar lesions may be considered in the differential diagnosis of calcified maxillary sinus lesions.

From a clinical perspective, this case emphasizes the need for careful evaluation of dental sources when calcification is found in a unilateral opacified maxillary sinus. If attention is focused only on the calcified appearance, the lesion may be interpreted as fungal sinusitis. However, when the lesion is located near a severely diseased maxillary tooth, odontogenic sinusitis with antrolith, sequestrum, isolated bone fragment, or inflammatory bone formation may also be considered. In such cases, panoramic radiography, computed tomography, intraoral examination, periodontal probing, and careful evaluation of the relationship between the lesion and adjacent teeth are all important. Recent multidisciplinary consensus statements on odontogenic maxillary sinusitis likewise emphasize that accurate diagnosis and successful management require identification of the odontogenic source, appropriate dental treatment, and close cooperation between dental and otolaryngologic specialists [2,5].

The treatment strategy in this case was appropriate because both the odontogenic source and the sinus lesion were managed simultaneously. Extraction of the maxillary left first molar eliminated the suspected source of chronic odontogenic inflammation, while removal of the calcified mass and maxillary sinus fenestration allowed drainage and resolution of sinus inflammation. At one-year follow-up, mucosal thickening of the left maxillary sinus had improved, and the patient had a favorable functional course with an obturator prosthesis.

This case has some limitations. Because it is a single case, the exact mechanism of bone formation cannot be proven. In addition, because this case occurred in 2011 and was reviewed retrospectively, some additional clinical details and long-term follow-up data beyond one year were not available. A coronal CT image of sufficient quality for publication was also unavailable. Furthermore, KOH preparation and fungal culture were not performed; therefore, evaluation for fungal disease was based on histopathological examination rather than microbiological confirmation. The lesion was diagnosed after it had already become necrotic, and the temporal sequence of inflammation, bone formation, separation, and sequestration could not be directly observed. Nevertheless, the clinical, radiographic, intraoperative, and histopathological findings collectively support the interpretation that chronic odontogenic maxillary sinusitis was associated with formation of a sequestrum-like bone mass.

In summary, this single case illustrates that calcified material within an opacified maxillary sinus may have a non-fungal origin and may radiographically mimic fungal sinusitis. In the present case, the lesion’s proximity to a severely diseased maxillary molar, along with the intraoperative and histopathological findings, supported a diagnosis of odontogenic maxillary sinusitis associated with a sequestrum-like bone mass. Therefore, when a calcified or bone-like lesion is observed near a diseased maxillary tooth, odontogenic maxillary sinusitis-associated lesions and other calcified or osseous lesions, including antrolith, isolated bone fragment, ectopic bone formation, osteoma, or sequestrum, may be considered in the differential diagnosis. When imaging findings are inconclusive and surgical treatment is clinically indicated, lesion removal and histopathological examination can help establish a definitive diagnosis.

## Conclusions

We reported a case of chronic odontogenic maxillary sinusitis associated with a sequestrum-like bone mass that radiographically mimicked fungal maxillary sinusitis. Although calcification or hyperdensity within an opacified maxillary sinus is an important clue for fungal sinusitis, this case illustrates that similar radiographic findings may also occur in odontogenic maxillary sinusitis with sequestrum-like bone formation. In this patient, the close relationship between the calcified lesion and the diseased maxillary molar, together with the intraoperative and histopathological findings, supported an odontogenic origin. Therefore, when a calcified or bone-like lesion is observed near a diseased maxillary tooth, odontogenic maxillary sinusitis-associated calcified or osseous lesions, such as sequestrum, antrolith, or isolated bone fragment, may be considered in the differential diagnosis. Multidisciplinary evaluation by dental and otolaryngologic specialists is important for correlating dental findings with sinonasal imaging. When surgical treatment is clinically indicated, removal of the odontogenic source and the lesion, followed by histopathological examination, can help establish a definitive diagnosis and guide appropriate management.
